# Potential of big data approach for remote sensing of vehicle exhaust emissions

**DOI:** 10.1038/s41598-021-84890-7

**Published:** 2021-03-09

**Authors:** Lijun Hao, Hang Yin, Junfang Wang, Xiaohu Wang, Yunshan Ge

**Affiliations:** 1 School of Mechanical Engineering, Beijing Institute of Technology, Beijing, 100081 China; 2 State Environmental Protection Key Laboratory of Vehicles Being Driven on the Road Emission Control and Simulation, Chinese Research Academy of Environmental Sciences, Beijing, 100012 China; 3 Anhui Baolong Environmental Protection Technology Co., Ltd., Hefei, 230031 China

**Keywords:** Environmental sciences, Engineering

## Abstract

At present, remote sensing (RS) is applied in detecting vehicle exhaust emissions, and usually the RS emission results in a definite vehicle specific power (VSP) range are used to evaluate vehicle emissions and identify high-emitting vehicles. When the VSP exceeds this range, the corresponding vehicle emission RS data will not be used to assess vehicle emissions. This method is equivalent to setting only one VSP Bin qualified for vehicle emission evaluation, and generally only one threshold limit is given for each emission pollutant without considering the fluctuation characteristics of vehicle emissions with VSP. Therefore, it is easy to cause misjudgment in identifying high-emitting vehicles and is not conducive to scientific management of vehicle emissions. In addition, the vehicle emissions outside the selected VSP Bin are more serious and should be included in the scope of supervision. This research proposed the methods of vehicle classifications and VSP Binning in order to categorize the driving conditions of each kind of vehicles, and a big data approach was proposed to analyze the vehicle emission RS data in each VSP Bin for vehicle emission evaluation.

With the continuous growth of vehicle population, vehicle emissions have become the main source of air pollution. By the end of 2019, the number of motor vehicles in China has reached 348 million, of which the number of automobiles was 260 million. The total emissions of four exhaust pollutants released from motor vehicles are 16.038 million tons, of which carbon monoxide (CO), hydrocarbons (HC), nitrogen oxides (NOx), and particulate matter (PM) emissions are 7.716 million tons, 1.892 million tons, 6.356 million tons and 74,000 tons respectively^1^. Automobiles are the main contributor to motor vehicle emissions, among which the CO and HC emissions of gasoline vehicles exceed 80% and 70% of the total vehicle emissions respectively, and the NOx and PM emissions from diesel vehicles contributed more than 80% and 90% of the total vehicle emissions^1^. Therefore, the emission control of gasoline vehicles mainly focusses on gaseous emission pollutants of CO, HC and NOx, while that of diesel vehicles concentrates on NOx and PM emissions.

Although the emission standards for new vehicles are continuously tightened, after the new vehicles are put into use, their exhaust emissions will gradually deteriorate due to wear and deterioration of auto parts^2^. Therefore, the Inspection and Maintenance (I/M) programs are essential to control vehicle emissions, and of great significance to improving air quality.

At present, the regular inspection methods for gasoline vehicle exhaust emissions mainly adopt acceleration simulation mode (ASM), vehicle emission mass analysis system (VMAS) or the two-speed idle method; For in-use diesel vehicle emission inspection, the lug-down test is mainly used to measure NOx and exhaust smoke emissions, or free acceleration test is utilized to test exhaust smoke opacity when the test conditions for lug-down cannot be met. At present, China has enforced regular inspections of vehicle emissions across the country. By the end of 2019, a total of 247,808,800 automobiles participated in legislative emission inspection across the country accounting for 98.1% of the total automobiles. According to statistics, the average pass rate of legislative emission inspections for in-use vehicles in China is about 90%, and about 10% of vehicles, which exceed the in-use vehicle emission standards, have a high contribution to the total vehicle emissions^1^. The vehicles that do not meet the emission inspection standards must be repaired to meet the in-use vehicle emission standards, otherwise they are forbidden to drive on road.

Although the legislative periodical emission inspections for in-use vehicles can effectively detect high-emission vehicles, the period of legislative emission inspections for in-use vehicles generally ranges from 6 months to 2 years, vehicle emissions may deteriorate before the next scheduled inspection. Therefore, there is an urgent need for a more convenient, fast and effective emission detection method for in-use vehicles, vehicle emission remote sensing technique can realize real-time monitoring and make up for the deficiencies of the regular vehicle I/M rules.

The RS technology for vehicle emission measurement originated in the late 1980s. In 1989, researchers at the University of Denver used non-dispersive infrared (NDIR) remote sensing equipment to measure vehicle CO emission^3^. Later, the remote sensing device (RSD) for the measurement of vehicle CO, HC and NO emissions was developed and put into use^4^.

The infrared light (or laser) or ultraviolet light beams emitted by RSD pass through the vehicle exhaust plume and will be partially absorbed, then measured by the RS system. By measuring and processing the spectral changes of the light beams, the concentration ratios of CO, HC, NO to carbon dioxide (CO_2_) in the vehicle exhaust plume and their absolute concentrations in tail pipe can be calculated^3–5^. At the same time, the vehicle speed and acceleration are measured, the vehicle license plate is recorded, and the environmental parameters are also monitored and recorded by the meteorological instrument. Finally, the main control computer or the vehicle emission monitoring system evaluates the vehicle emission level and identifies high-emitting vehicles.

In the US, the states of Texas, Missouri and Colorado have successively used vehicle emission remote sensing data for the exemption of clean vehicles or screening of high-emitting vehicles^6–8^, Other countries and regions, such as Mexico, New Zealand, and the United Kingdom, also implemented RSD for vehicle emission measurement^9,10^.

Many cities in China including Beijing, Guangzhou, Chongqing, Shanghai, Hong Kong, etc. have introduced RSD to measure vehicle emissions for the sake of identifying high-emitting vehicles driven on the road^11,12^. By the end of 2019, 2671 sets of RSDs have been applied nationwide. According to statistics, 359.084 million RS data measurements of vehicle emissions were recorded and accumulated in 2019, among them 10.8979 million emission RS data were found to exceed the standard limits, accordingly the capture rate of high-emitting vehicles accounted for 3.03%.

Based on China's plan of building vehicle emission remote sensing network^1^, each of 350 regional level cities will build at least 10 RSDs, that means more than 3500 sets of RSDs will be utilized in China, and there will be an explosive growth of vehicle emission RS data in the future.

Remote sensing of gasoline vehicle emissions has been applied for nearly 30 years since the researchers at University of Denver measured the vehicle CO emission using remote sensing^3^. Until now, the application of RSDs has achieved good results for measurements of CO, HC and NO concentration emissions from gasoline vehicles, but has a high false rate in detecting the tailpipe emission concentrations for diesel vehicles^13^. Because the vehicle emissions variation characteristics with driving conditions have not been fully considered when establishing the evaluation criteria for high-emission vehicles. At present, vehicle specific power (VSP), which is the instantaneous vehicle tractive power per unit mass, is usually used as an important parameter to characterize vehicle driving conditions. Only within a defined VSP range, for example VSP values between 5 and 20 kW Mg^−1^, the emission RS data are used for evaluation of vehicle emissions and screening of high-emitting vehicles^14,15^. Outside this VSP window, the emission RS data are regarded as invalid data in order to avoid the variability of measurement results. Indeed, vehicle emissions outside this specified VSP range may be more serious and should be included in the scope of vehicle emission evaluation and supervision. And within this specified VSP window usually one emission threshold limit is given for each emission pollutant for screening high-emission vehicles ignoring the varying characteristics of vehicle emissions with VSP. This treatment method is easy to cause misjudgment of high-emission vehicles and not helpful for scientific management of vehicle emissions, and also reduce the effectiveness of the vehicle emission remote sensing system.

In order to make full use of the vehicle emission RS data, this paper investigated a big data analysis approach for evaluation and supervision of vehicle emissions based on the RS data of vehicle exhaust emissions, and determined the threshold limits of each exhaust pollutant for identifying high-emission vehicles within different VSP windows.

## Results

### Big data approach for analyzing the RS data of gasoline vehicle exhaust emissions

The speed and torque of gasoline engine change under different vehicle driving conditions, consequently, the amount of air fuel mixture needed for each working cycle and its combustion performance of gasoline engine vary significantly. Therefore, gasoline vehicle exhaust emissions including CO, HC and NO are closely related to driving conditions, which can be characterized by VSP.

**Figure 1:**
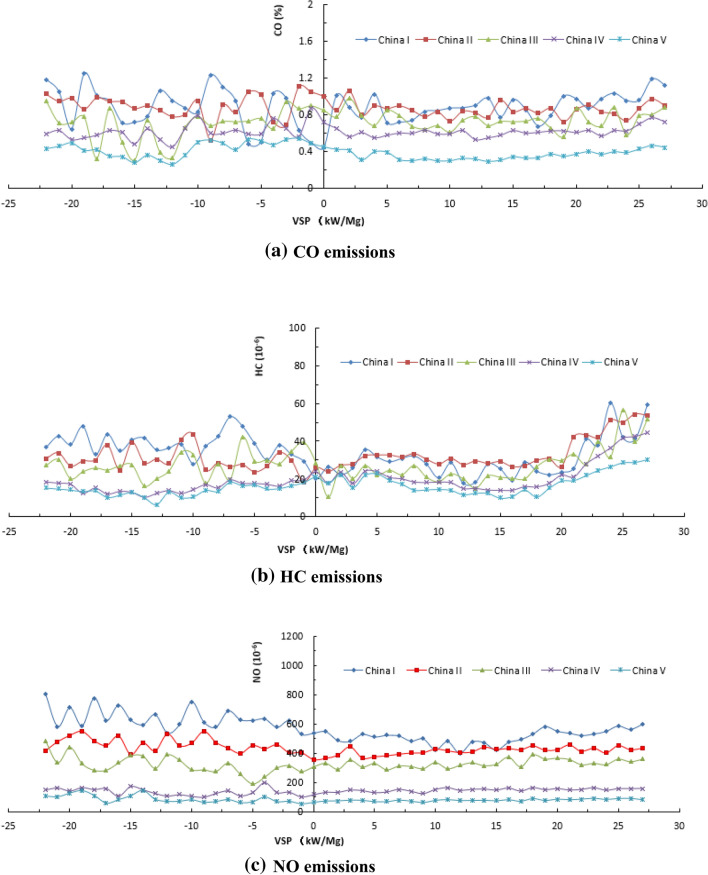
The relationship between the RS emissions and VSP of light-duty gasoline vehicles.

The emission RS data of some light-duty gasoline vehicles in Beijing were statistically analyzed as a function of VSP. Considering that China VI emission standard has just been implemented in China, and there are few emission RS data of China VI light-duty gasoline vehicles, this research statistically analyzed the emission RS data of light-duty gasoline vehicles compliant with China I, China II, China III, China IV and China V. The emission characteristics of CO, HC and NO emitted from light-duty gasoline vehicles are shown in Fig. 1. The CO, HC, and NO emissions of light-duty gasoline vehicles at each emission stage are relatively stable in the VSP range between 0 and 20 kW Mg^−1^, which is defined as the valid VSP window for RS emission detection required by local regulations in Beijing, Anhui, Shandong, etc. Most of the emission RS data beyond this VSP range are abnormally high values, so it is not used for subsequent vehicle emission evaluation based on the current vehicle emission RS regulations.

**Figure 2:**
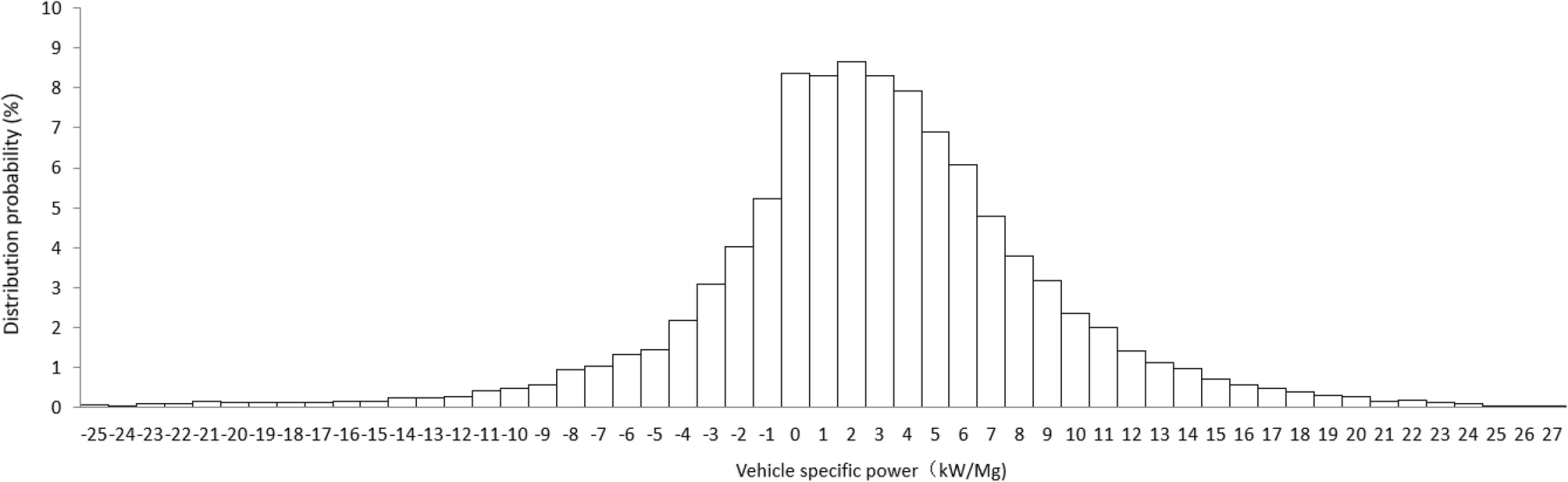
VSP distribution probability of light gasoline vehicles in Beijing.

In order to analyze the percentage of emission RS data within the defined VSP range used for vehicle emission evaluation in the whole measurement data, the probability distributions of the VSP values for light-duty gasoline vehicles in Beijing were statistically analyzed and shown in Fig. 2.

As shown in Fig. 2, the highest probability distribution density of VSP is in the range of 0–5 kW Mg^−1^ for light-duty gasoline vehicles. Outside this range, as the VSP increases or decreases, the occupation probabilities of the vehicle emission RS data decrease. It can be seen from Fig. 2 that the amount of RS data in the range of 0–20 Mg^−1^ accounts for 76.8% of the total measurement data, while the amount of RS data in the range of 5–25 Mg^−1^, which is defined by US EPA, only accounts for 35.8% of the total data. Using one VSP Bin and usually only one emission limit given for each pollutant does not consider the variation characteristics of vehicle emissions with VSP, and is easy to cause misjudgment in identifying high-emission vehicles. Since the valid VSP range defined for remote sensing of vehicle emissions is only a limited VSP section, beyond this range the emission RS data will not be used for subsequent vehicle emission evaluation, which causes plenty of emission RS data to be invalid and cannot make full use of the remote sensing system in evaluating vehicle emissions. In addition, vehicle emissions outside the recommended VSP range used for vehicle emission evaluation may be more serious and should be included in the scope of vehicle emission assessment.

Considering the differences of gasoline vehicle emission characteristics under different driving conditions, a big data approach was proposed to statistically analyze emission RS data based on gasoline vehicle classifications and their VSP partitions in order to realize scientific and refined management of vehicle emissions.

Gasoline vehicles can be classified into different categories. In this research, they were categorized into light-duty vehicles and heavy-duty vehicles based on the current emission standards^16^. Furthermore, the light-duty gasoline vehicles can be further classified into passenger cars, delivery vans, light-duty trucks, and minibuses. Similarly, the heavy-duty vehicles can be classified as big buses, distribution vehicles and heavy-duty trucks. Few gasoline engines are used for heavy-duty vehicles, they are usually natural gas engines, and methanol or ethanol fueled engines. The methods of vehicle classification and data processing in this research are applicable to other spark ignition engine vehicles.

The VSP binning method for light-duty gasoline vehicles is presented in Table 1, which shows the VSP range of light-duty gasoline vehicles is divided into 20 Bins, whose width depend on the VSP value and the probability distributions of vehicle emission RS data in them. The VSP Bin that has less emission RS data is assigned into a wider VSP range. The Bin partitions of VSP can be adjusted in time based on the amount of emission RS data and the need for refined management of vehicle emissions.

**Table 1:** Bin partitions of VSP for light-duty gasoline vehicles.

No. of bin	VSP bin/(kW Mg^−1^)	No. of bin	VSP bin/(kW Mg^−1^)
1	VSP < − 12	11	4 ≤ VSP < 5
2	− 12 ≤ VSP < − 7	12	5 ≤ VSP < 6
3	− 7 ≤ VSP < − 4	13	6 ≤ VSP < 7
4	− 4 ≤ VSP < − 2	14	7 ≤ VSP < 8
5	− 2 ≤ VSP < − 1	15	8 ≤ VSP < 9
6	− 1 ≤ VSP < 0	16	9 ≤ VSP < 10
7	0 ≤ VSP < 1	17	10 ≤ VSP < 12
8	1 ≤ VSP < 2	18	12 ≤ VSP < 14
9	2 ≤ VSP < 3	19	14 ≤ VSP < 17
10	3 ≤ VSP < 4	20	17 ≤ VSP

The remote sensing system detects the vehicle speed and acceleration, calculates the VSP of the tested vehicle under the test condition, and assigns the vehicle emission RS data to the corresponding Bin section based on the VSP value. In each Bin section, the vehicle emission RS data will be statistically analyzed for evaluation of vehicle emissions.

In each Bin of VSP, each emission RS data will be regarded as a discrete random variable, the probability distribution of each emission RS data will be statistically calculated, and furthermore the cumulative probabilities of the emission RS data are also calculated. According to the recommended screening ratio of high-emitting vehicles, for example 5%, the vehicles whose emission RS data are within the cumulative distribution probability of 95% can be regarded as emission-qualified. Then the x-axis data which is corresponding to the cumulative distribution probability of 95% is used as the emission threshold limit for screening high-emitting vehicles.

**Figure 3:**
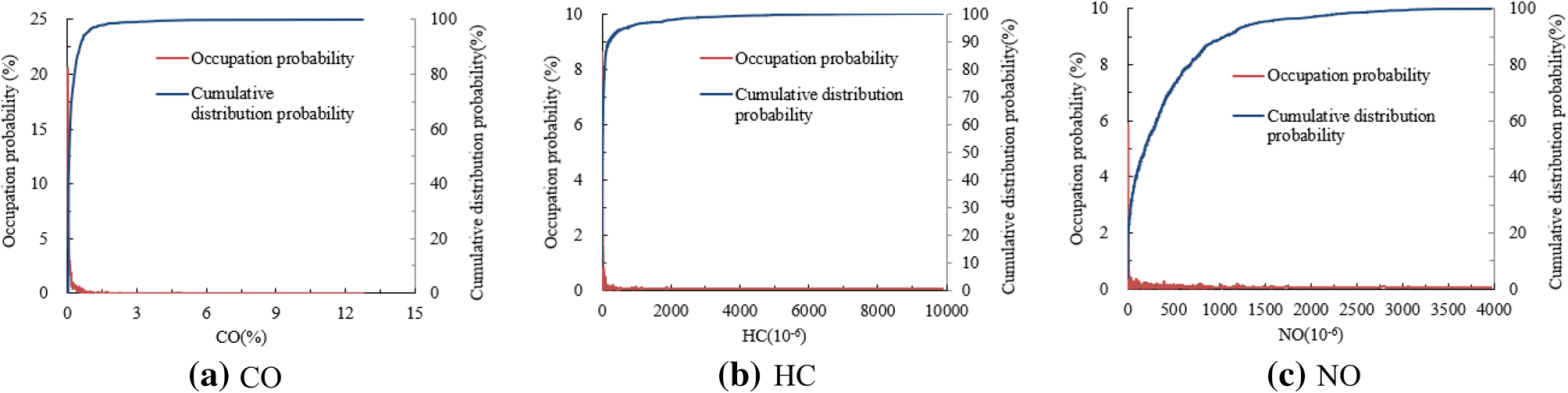
Probability distribution and cumulative distribution probability of RS data of CO, HC and NO emissions from light-duty gasoline vehicles tested under ASM5024.

The emission RS data of light-duty gasoline vehicles tested under BASM5024, which is used for the regular emission inspection of light-duty gasoline vehicles in Beijing using the test speed of 24 km h^−1^, were used as an example for statistical analysis. The VSP of light-duty gasoline vehicles is about 5.8 kW Mg^−1^ under Beijing ASM5024 test, therefore the emission RS data are allocated in the VSP Bin12 and statistically analyzed. The probability distribution and cumulative distribution probability of RS data of CO, HC and NO emissions are shown in Fig. 3. The statistical analysis of the RS emission data is aimed to obtain the emission RS data threshold limit for screening high-emitting vehicles, therefore the emission stages of light-duty gasoline vehicles need not be distinguished in order to identify high-emitting vehicles.

The emission RS data and statistically analyzed emission results allocated in each VSP Bin are always dynamically updated. In order to facilitate data analysis, the light-duty gasoline vehicle emission RS data in VSP Bin 12 were temporarily regarded as static to illustrate the big data analysis method, and analyzed to determine the screening threshold limit for identifying high-emission vehicles.

Nowadays, for RS detection of vehicle emissions, the capture rate is about 5% for the high-emitting vehicles, that means approximately 5% of the vehicles driven on road are identified as high-emitting vehicles. Considering each emission pollutant separately, the tested emission data, which corresponds to the cumulative distribution probability of 95% for each emission pollutant, is regarded as the cutpoint of emission RS data for screening high-emitting vehicles. The cutpoints of HC, CO and NO concentrations for screening high-emitting vehicles under ASM5024 test are statistically analyzed and shown in Table 2.

**Table 2:** Analysis of CO, HC and NO emissions from light-duty gasoline vehicles tested under ASM5024.

Emissions	Maximum of raw data	Statistical average value of raw data	Cutpoints of emission concentrations for screening high-emitting vehicles	Statistical average value after screening out high emission vehicle data
CO (%)	12.77	0.23	0.82	0.14
HC (10^−6^)	9886	169.26	735	47.7
NO (10^−6^)	3968	396.62	1425.2	302.48

**Figure 4:**
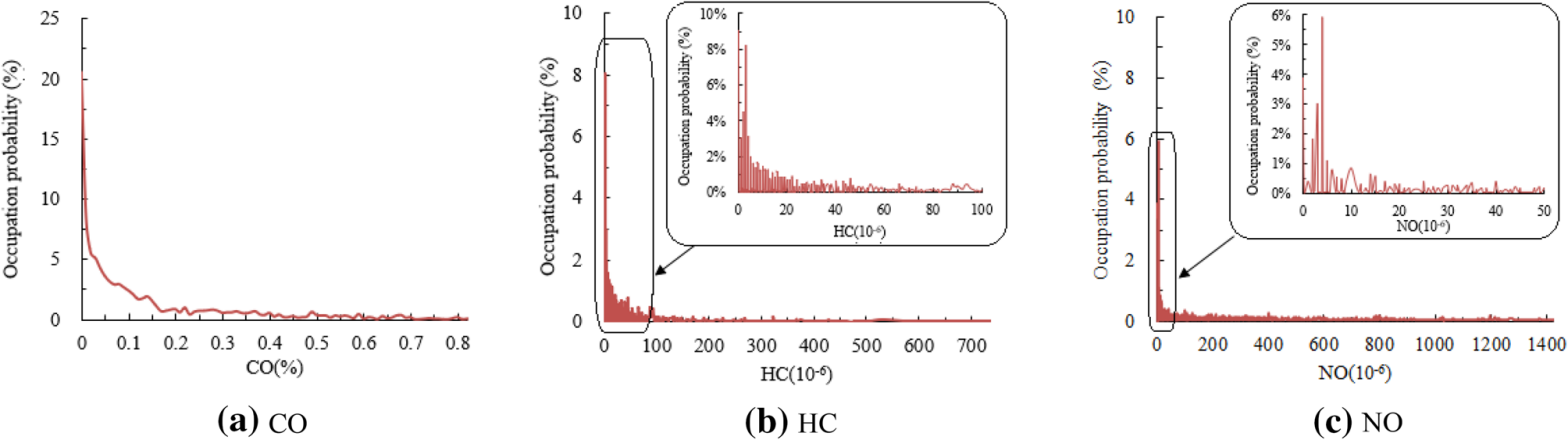
Probability distribution of emission data after excluding 5% high-emitting vehicles.

As shown in Table 2, each emission component is taken as the control target alone and processed separately. For the capture rate of 5% high-emitting vehicles, the CO emission RS screening threshold is 0.82% for detecting high-emitting vehicles. If the detected high-emitting vehicles are repaired and meet the emission standards, their emission data will be deleted from database in that Bin, therefore the maximum value of CO emission RS data in the Bin is reduced from the maximum value of 12.77 to 0.82%, the statistical average of CO emission data reduced from 0.23 to 0.14%. After screening out the 5% high-emitting vehicles, the probability distribution of CO emission RS data is shown in Fig. 4a.

Treated in the same way, the RS emission screening thresholds of HC and NO for detecting high-emitting vehicles are 735 × 10^−6^ and 1425.2 × 10^−6^ respectively. After excluding the 5% high-emitting vehicles whose emissions have been improved completely, the maximum values of HC and NO emissions are reduced from 9886 × 10^−6^ and 3968 × 10^−6^ to 735 × 10^−6^ and 1425.2 × 10^−6^ respectively, and the statistical average values of HC and NO emission data are reduced from 169.26 × 10^−6^ and 396.62 × 10^−6^ to 47.7 × 10^−6^ and 302.48 × 10^−6^ respectively. After screening out the 5% high-emitting vehicles, the probability distributions of HC and NO emission RS data are shown in Fig. 4b, c.

Since each vehicle emission RS results are a set of data (CO, HC and NO), and the CO, HC and NO emissions of one vehicle may exhibit different emission characteristics, maybe only one pollutant exceeds the emission threshold limit, and the other two not. Or two exhaust pollutants exceed the emission thresholds and one not, or all three exhaust pollutants exceed the threshold limits. Therefore, if one of the three vehicle exhaust pollutants such as CO, HC or NO exceeds the emission threshold, the vehicle will be identified as high-emitting vehicle, the actual screening ratio of high-emitting vehicles in the total number of tested vehicles will be far greater than 5%; and if the high-emitting vehicle is confirmed only when all its three emission pollutants exceed their emission threshold limts, the actual screening ratio of high-emitting vehicles in the vehicle fleet will be less than 5%. Therefore, it is necessary to adjust the screening rate for each emission pollutant in order to guarantee the final screening rate of high-emitting vehicles around 5%.

### Big data approach for analyzing the RS data of diesel vehicle exhaust emissions

The remote sensing of diesel vehicle exhaust emissions focuses on NO emission and exhaust smoke. The methods of vehicle type classification, VSP binning and emission RS data analysis for diesel vehicles are the same as that of gasoline vehicles. Since the NO and smoke emissions of diesel vehicles are mainly affected by engine load, NO and smoke emissions tend to increase when the load of diesel engine increases. On the other hand, when diesel vehicles decelerate, the diesel engine enters idling and may adopt fuel cutoff strategy, the NO and smoke emissions decrease. Therefore, the test conditions for remote sensing of diesel vehicle exhaust emissions require that vehicle acceleration or VSP is greater than or equal to 0, otherwise the diesel vehicle emission RS results can be ignored. The VSP binning method for light-duty diesel vehicles is presented in Table 3, which shows the VSP range of light-duty diesel vehicles is divided into 14 Bins, which can also be adjusted in time based on the amount of emission RS data and the need for refined management of vehicle emissions.

**Table 3:** Bin partitions of VSP for light-duty diesel vehicles.

No. of bin	VSP bin/(kW Mg^−1^)	No. of bin	VSP bin/(kW Mg^−1^)
1	0 ≤ VSP < 1	8	7 ≤ VSP < 8
2	1 ≤ VSP < 2	9	8 ≤ VSP < 9
3	2 ≤ VSP < 3	10	9 ≤ VSP < 10
4	3 ≤ VSP < 4	11	10 ≤ VSP < 12
5	4 ≤ VSP < 5	12	12 ≤ VSP < 14
6	5 ≤ VSP < 6	13	14 ≤ VSP < 17
7	6 ≤ VSP < 7	14	17 ≤ VSP

At present, same as gasoline vehicles, the remote sensing regulations for diesel vehicle emissions usually apply a single threshold for each exhaust pollutant in identifying high-emission vehicles over all driving conditions. It is easy to cause misjudgment because the high-emitting vehicles may pass emission test due to low emissions when driving at low load or low speed. On the other hand, some low-emission vehicles may be misjudged as high-emitters due to their higher emissions under high loads. In order to solve this problem, the big data approach is also used for remote sensing of diesel vehicle emissions.

The VSP of diesel vehicle can be calculated based on the measured speed and acceleration, and the VSP range can be binning as the same way as gasoline vehicles. Then the NO emission and exhaust smoke data are distributed to a certain VSP Bin based on the calculated VSP for statistical analysis.

**Figure 5:**
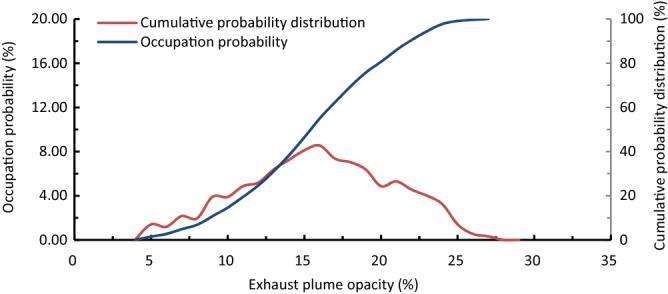
Probability distribution and cumulative distribution probability of RS exhaust opacities of light-duty diesel vehicles in Bin5.

As the RS technique for measuring absolute concentration of NO emission from diesel vehicles is still being investigated and verified^17^, there is no sufficient amount of RS data of NO emission from diesel vehicles so far. Therefore, in this paper, the RS smoke opcities of light-duty diesel vehicles are used for statistical analysis of the cutpoint used to identify the high-emission vehicles. Selecting the RS data of light-duty diesel vehicle exhaust smoke in Bin5 (4 kW Mg^−1^ ≤ VSP < 5 kW Mg^−1^) as an example for analysis, the RS smoke opacities of diesel vehicles are statistically analyzed. The probability distribution and cumulative distribution probability of the RS smoke opacities are shown in Fig. 5, and the screening threshold of exhaust opacity for detecting high-emitting vehicles in Bin5 is analyzed, as shown in Table 4.

**Table 4:** Screening threshold of exhaust opacity for detecting high-emitting vehicles in Bin5.

Emission	Raw data of exhaust smoke opacity in Bin5		Cutpoints of exhaust smoke opacity for screening high-emitting vehicles in Bin5	Statistical average data after screening out 5% high emission vehicles
	Maximum	Statistical average		
Opacity (%)	27	15.88	23.5	14.52

For the convenience of analysis, the exhaust opacity RS data are also temporarily processed as static. For the screening rate of 5% high-emission diesel vehicles, the exhaust opacity threshold is 23.5%. The diesel vehicle will be recognized as a high-emitter if its RS exhaust opacity exceeds 23.5% for the number of times required by RS regulations in Bin5. If the detected high-emitting vehicles meet the emission standards after repair, the RS data of the 5% high-emitting vehicles will be deleted from the RS data base of this Bin, then the maximum exhaust opacity in the VSP Bin reduced from 27 to 23.5%, and the statistical average of the exhaust opacity RS data dropped from 15.88 to 14.52%.

### Monitoring of vehicle exhaust emissions by means of remote sensing big data methodology

Since the magnitude of vehicle emissions are affected by seasonal environmental conditions, such as atmospheric pressure, temperature and humidity. Statistical analysis of vehicle emission RS data can be carried out across the country by different regions.

Based on the central limit theorem, when the number of samples is greater than 30, the central limit theorem can work. Therefore, for statistical analysis of remote sensing measurements in each VSP bin, much larger samples are more beneficial for the big data analysis.

In each VSP Bin, each vehicle emission pollutant will be assigned a counter to count the number of times that its RS result exceeds the screening threshold limit for high-emitting vehicles, and all the counters of the same emission pollutant allocated in all VSP Bins will be added up to calculate the number of records exceeding emission limit, which will be compared with the specified number of times within the defined time period, for example, two times within 6 months defined by the HJ 845-2017 standard^18^. For any one of vehicle emission pollutants, if its number of times exceeding the screening threshold limit surpass the required number of times specified by RS regulations, the vehicle will be determined as a high-emission vehicle, and the owner is notified to check and repair the vehicle.

After the high-emitting vehicle is repaired and its emissions meet the emission standards, its RS emission data in the database will be deleted, but its history record of exceeding emission limit will be kept. The vehicle emission monitoring system regularly counts the history records of exceeding emission limits for all vehicle types, and evaluate the emission levels of all vehicle models on the market, and focuses on the implementation of emission inspections and emission supervision for the vehicle types with a relatively high failure rate.

As the amount of vehicle emission RS data increases, the emission RS data in each Bin section are always updated and statisticaly analyzed in real time, a certain percentage (for example 5%) of high-emitting vehicles can always be screened out and improved by mandatory maintenance or scrapping. The average emissions of the overall vehicle fleet should continue to reduce due to continuous screening of high-emission vehicles. Over time, the number of low-emission vehicles increase, old vehicles are scrapped, and the composition of vehicle fleet changes. As the vehicle emission RS data in each VSP Bin are updated in real time, the RS emission screening thresholds for identifying high-emitting vehicles are also dynamically updated with the change of the vehicle fleet composition. This has the incomparable superiority of real-time updating compared with the in-use vehicle emission standards because the adjustment of the in-use vehicle emission limits is restricted by the time and process of emission standard formulation and revision.

The statistical average of the emission RS data in each VSP Bin is the weighted average of all the data in the Bin section. Not only the value of each emission RS data is considered, but also the probability of its value. As time accumulates, the number of emission RS data stored in each Bin increases rapidly. Due to the application of big data approach, the huge number of RS data is no longer a difficulty or obstacle to solving the problem, but instead becomes a guarantee that the statistical average of the emission RS data in each Bin has practical significance and excellent robustness. The statistical average emission value of each Bin is enough to represent the true value of the vehicle emission in the Bin section, and can be used for vehicle emission level evaluation and emission estimation. The impact of any emission RS result on the statistical average emission of each Bin is so small that it can be ignored, and the average vehicle emission level characterized by the statistical average emissions of each Bin is synchronized with the change of the composition of vehicle fleet driven on the road.

## Method

### Inversion calculation method for remote sensing results of gaseous emissions from gasoline vehicles

Since vehicle exhaust gas is diluted by the ambient air, the concentrations of various emission components measured by RSD in the exhaust plume are not the true emission concentrations of vehicle exhaust. The processing method of RSD takes CO_2_ as the reference gas, and regards the concentration ratios of CO, HC, NO to CO_2_ as constant values. By measuring the volume concentration ratios of CO, HC and NO to CO_2_, the actual emission concentration of CO_2_ in the gasoline vehicle exhaust is calculated based on theoretical air–fuel mixture combustion^3–5^.

1 $$C_{{{\text{CO}}_{2} }} = \frac{42}{{2.79 + 2\emptyset_{{{\text{CO}}}} + 1.21\emptyset_{{{\text{HC}}}} + \emptyset_{{{\text{NO}}}} }}$$

where $\emptyset_{{{\text{CO}}}}$, $\emptyset_{{{\text{HC}}}}$ and $\emptyset_{{{\text{NO}}}}$ are the volume concentration ratios of CO, HC and NO to CO_2_ in the exhaust plume, respectively.

Therefore the absolute volume concentrations of CO, HC and NO emitted from gasoline vehicles can be calculated by

2 $$C_{{{\text{CO}}}} = C_{{{\text{CO}}_{2} }} *\emptyset_{{{\text{CO}}}}$$

3 $$C_{{{\text{HC}}}} = C_{{{\text{CO}}_{2} }} *\emptyset_{{{\text{HC}}}}$$

4 $$C_{{{\text{NO}}}} = C_{{{\text{CO}}_{2} }} *\emptyset_{{{\text{NO}}}}$$

The RSD measures the speed and acceleration of the tested vehicle, and calculates its VSP under the tested condition. VSP is commonly used as a parameter to comprehensively assess the influences of vehicle speed and acceleration on vehicle emissions^19^, and can be calculated by

5 $$VSP = \left[ {\frac{{C_{D} A_{f} }}{{m_{v} }}\frac{{\rho_{a} }}{2}\left( {v \pm v_{w} } \right)^{2} + gf_{R} \cos \varphi + a\left( {1 + \varepsilon } \right) + g\sin \varphi } \right]v$$

where ${ }C_{D}$ is air drag coefficient, $A_{f}$ is vehicle frontal area (m^2^), $\rho_{a}$ is air density, $v$ is vehicle speed (m s^−1^), $v_{w}$ is headwind speed (m s^−1^), $g$ is gravity acceleration, $f_{R}$ is the coefficient of rolling resistance, $\varphi$ is the angle of the roadway with the horizontal, $a$ is vehicle acceleration (m s^−2^), $\varepsilon$ is mass factor(representing the equivalent ratio of translational mass of the rotating components), and $m_{v}$ is vehicle mass (Mg).

The emission RS data will be allocated into the corresponding VSP Bin according to the VSP value^21^.

### Inversion calculation method for remote sensing emissions from diesel vehicles

Formulas 1–4 are not applicable for remote sensing of gaseous emissions from diesel vehicles due to the lean combustion of diesel engine. The excessive air coefficient of diesel engine under the test condition must be considered^17^, and the inversion calculation method of absolute concentration of CO_2_ in the diesel vehicle exhaust is calculated by:

6 $$C_{{{\text{CO}}_{2} }} = \frac{100}{{0.5\emptyset_{{{\text{HC}}}} - 0.5 + 2.38\upalpha (2\emptyset_{{{\text{CO}}}} + \emptyset_{{{\text{HC}}}} + 3 + \emptyset_{{{\text{NO}}}} )}}$$

where α is the excessive air coefficient of the diesel engine combustion under the test condition of the diesel vehicle.

The absolute concentrations of CO, HC and NO emissions released from diesel vehicle can also be calculated using the formulas (2), (3) and (4).

Therefore, for remote sensing of gaseous emissions from diesel vehicles, not only the relative concentration ratios of NO, CO, HC to CO_2_ in the exhaust plume need to measured, but also the excessive air coefficient of diesel engine combustion under the test condition must be obtained.

In order to calculate the diesel engine excessive air coefficient, it is necessary to convert the motive force and speed of the diesel vehicle into the engine torque and revolution speed, and calculate the excessive air coefficient of the engine under test conditions based on the diesel engine performance characteristics^17^. Another way of characterizing the diesel engine excessive air coefficients is to set up the statistical map of diesel engine excessive air coefficients as a function of diesel vehicle speed and acceleration, and use the tested vehicle speed and acceleration to interpolate the excessive air coefficient map to obtain the engine excessive air coefficient under the test condition^20^.

The exhaust smoke of diesel vehicle is detected by photoelectric sensors, and the maximum smoke opacity will be recorded.

### Big data approach for analyzing vehicle emission RS results

Because remote sensing typically records a short time snapshot of a vehicle’s exhaust emissions, only one or several results for each exhaust component may be obtained when performing remote sensing test on vehicle emissions. In each Bin of VSP, the emission RS data are regarded as discrete random variables, their probability distributions and cumulative distribution probability are analyzed^21,22^.

The probability distribution of the discrete emission RS data $x_{i}$ can be expressed as

7 $$P\left( {x_{i} } \right) = p_{i}$$

Therefore, the probability $p_{i}$ satisfies the following equation.

8 $$\mathop \sum \limits_{i = 1}^{n} p_{i} = 1$$

The cumulative distribution function $f\left( x \right)$ of the discrete emission RS variable $x_{i}$ is shown as below.

9 $$f\left( {x_{i} } \right) = \mathop \sum \limits_{1}^{i} p_{i}$$

The cumulative distribution probability function value ${\text{f}}\left( {x_{i} } \right)$ represents the cumulative probability of $x$ falling in the interval (0 ~ $x_{i}$).

The statistical average value of emission RS data in each Bin can be calculated as

10 $$\overline{x} = \mathop \sum \limits_{i = 1}^{n} x_{i} p_{i}$$

Therefore, the statistical average of the emission RS data in each VSP Bin is the weighted average of all data in the Bin section, and is enough to represent the true value of the vehicle emission in the Bin section, and can be used for vehicle emission level evaluation and emission estimation.

## Data Availability

The data that supports the findings of this study are available from the corresponding authors upon request.
